# Effects of HBV Genetic Variability on RNAi Strategies

**DOI:** 10.1155/2011/367908

**Published:** 2011-07-02

**Authors:** Nattanan Panjaworayan, Chris M. Brown

**Affiliations:** ^1^Department of Biochemistry, Faculty of Science, Kasetsart University, Bangkok 10900, Thailand; ^2^Department of Biochemistry, University of Otago, Dunedin 9016, New Zealand

## Abstract

RNAi strategies present promising antiviral strategies against HBV. RNAi strategies require base pairing between short RNAi effectors and targets in the HBV pregenome or other RNAs. Natural variation in HBV genotypes, quasispecies variation, or mutations selected by the RNAi strategy could potentially make these strategies less effective. However, current and proposed antiviral strategies against HBV are being, or could be, designed to avoid this. This would involve simultaneous targeting of multiple regions of the genome, or regions in which variation or mutation is not tolerated. RNAi strategies against single genotypes or against variable regions of the genome would need to have significant other advantages to be part of robust therapies.

## 1. RNA Interference as an Antiviral Strategy

RNA interference (RNAi) is a sequence-specific mechanism to downregulate gene expression. Several pioneering studies have demonstrated the effectiveness of using siRNAs for treating viral diseases caused by HIV, hepatitis C virus (HCV), and HBV [1–5].

Clinical trials with RNAi have now begun for several disorders, but challenges such as off-target effects, toxicity, and safe and efficient delivery methods have to be overcome before the widespread use of RNAi as a gene-based therapy [6, 7]. For hepatitis B virus (HBV) several approaches have been taken using various design and delivery strategies with good initial success (reviewed in [4, 5, 8, 9]) and some limitations [10–12].

Several studies have tested the effect of variability in HBV viral genomes on effectiveness of this antiviral strategy; see [7, 13, 14] and references therein. This paper will outline the RNAi pathway, current delivery methods, current RNAi design strategies, and the effects of variation on these strategies. 

## 2. The Mechanism of RNAi

RNAi is initiated by short double-stranded RNAs (dsRNAs) that lead to the sequence-specific inhibition of their homologous RNAs [15–17]. In the case of HBV, this includes the 3.6 kb pregenomic RNA (pgRNA), although some targets are within multiple overlapping viral RNAs. 

**Figure 1:**
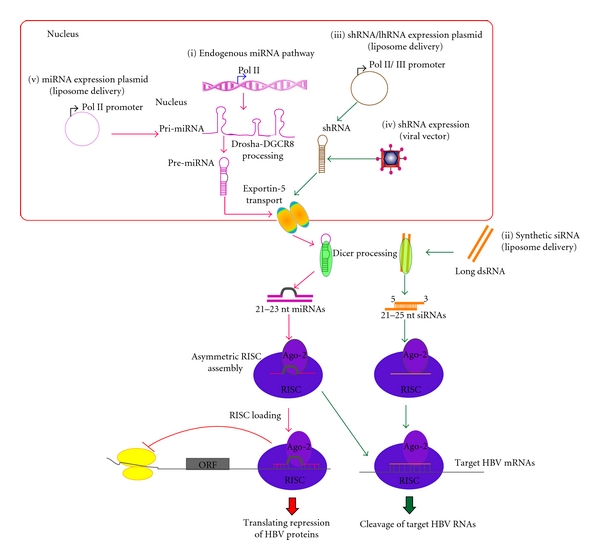
RNAi pathways in HBV research. Flow diagram of the miRNA pathway (i) is shown using red arrows, whereas the siRNA pathway is indicated using green arrows. Current RNAi strategies including delivery approaches (ii)–(v) are demonstrated.

Two major types of RNA have been channeled into the RNAi pathway small interfering RNAs (siRNAs) and microRNAs (miRNAs) by using synthetic dsRNAs or DNA vectors (Figure 1). The siRNAs have a characteristic two-nucleotide 3′ overhang, which are processed from larger dsRNAs by Dicer. They are incorporated into RISC, and the sense strand of the siRNA is removed [18–20]. Some studies using HBV have designed siRNAs (and miRNAs) to promote this asymmetric loading of the RISC complex. The antisense strand of the siRNA base pairs with its target RNA, with exact complementarity, and then RISC mediates cleavage and subsequent degradation of the target RNA [21–23] (Figure 1). Perfect base pairing between the siRNA and HBV RNA is a hallmark of siRNA effects, and single base substitutions in the target, due to genome variability, would disrupt this mode of action [4, 8, 17, 24].

Strategies based on miRNAs require engineering genes encoding longer primary transcripts (pri-miRNA based on miRNA genes) that are then processed into 60–70 base paired precursor miRNAs (pre-miRNAs) by the microprocessor complex [25, 26]. Following processing, the pre-miRNA is exported to the cytoplasm by the Ran-GTP-dependent cargo transporter Exportin-5 [27]. In the cytoplasm pre-miRNA is processed by Dicer into the mature miRNA, which is incorporated into RISC [4, 8, 17, 24] which targets the viral RNA [28]. Typical cellular miRNAs are not perfectly matched to their mRNA targets, and studies have indicated that they mainly exert silencing through translational repression, rather than degradation [29, 30] (Figure 1). However, later studies indicate that mismatched miRNA-mRNA duplexes can also trigger degradation [31, 32]. This may indicate that miRNAs targeted against the HBV pgRNA could also reduce levels of that RNA, rather than just its translation. 

## 3. RNAi Delivery Mechanisms

In order to use RNAi-based systems to target viral mRNAs, several delivery strategies have been developed. The two main current strategies are chemically synthesized siRNA duplexes and DNA-based expression cassettes that subsequently generate functional siRNAs in cells. These RNAs are usually short hairpin RNAs (shRNAs) or primary miRNAs (pri-miRNAs). 

Synthetic siRNA duplexes are usually delivered into cells via the endosomal pathway by cationic liposomes, whereas DNA-based expression cassettes require facilitating carriers such as liposomes or viral vectors (Figure 1). Synthetic siRNA duplexes have some limitations *in vivo*—rapid liver clearance, lack of target specificity, and expense [33–35]. To improve *in vivo* stability of siRNA duplexes, the backbone of siRNA may be chemically modified and linked to molecules such as 2′F, 2′O-Me, and 2H [36, 37]. 

DNA-based viral expression cassettes may provide cost-effective approaches for HBV treatment. Presently, there are a number of viral vectors under development. Each type of viral vector has specific characteristics that need to be determined for the specific target. The adenovirus- and adeno-associated virus- (AAV-) derived vectors provide an efficient delivery vehicle for transient shRNA expression [8]. Particularly, the Ad-gutless vector is used for liver-directed systemic delivery with prolonged silencing effects [38] while a conditionally replicating adenovirus (CRAd) is designed to replicate and kill tumour cells specifically [8]. Retroviruses on the other hand provide major advantage of incorporating the transgenic siRNA genes into the host cell genome for longer-term therapy [39]; other viral vectors have been used [40, 41]. 

## 4. Design of RNAi against HBV

To improve the efficiency of RNAi strategies and limit off-target effects, several research groups have improved the design of RNAi target sites. Certain characteristics of RNAi target sites contribute to siRNA efficiency; these have been utilised in some rational design approaches, whereas other studies have focused more on conservation of sites in HBV genomes. Specific features that should improve the efficiency of target sites include a UU overhang at the 3′-end [42, 43], a 30–50% GC content, which is effective for the unwinding of the duplex but sufficient for stabilizing interactions between siRNAs and their targets, and the nucleotide at the position 19 should preferentially be an adenine (A) base, as it is naturally found in miRNAs [8, 19, 20, 44]. Sun et al. report that there are about 170 sites in the HBV genome that meet simpler minimal criteria for RNAi design-target length 19, GC 35–60 and lack of homopolymer runs [7]. 

Other considerations relating specifically to RNA polymerase III (Pol III) transcription are that there should be no 4–6 base T tracts within the DNA sequence, because this could act as a termination signal [19]. Importantly, siRNAs must be specific to their target HBV mRNAs and have minimal similarity to cellular mRNA sequences, at least for RNAs expressed in the targeted cells (hepatocytes) to avoid off-target effects.

Results from McCaffrey and Ely et al. indicate that miRNA-based RNAi effectors against HBV pregenomic RNA were more effective than shRNA-based RNAi effectors for the same target sites [8, 24]. Grimm et al. [11] found that the RNAi toxicity may be caused by competition between the exogenous expressed shRNA and endogenous miRNA for the RNAi machinery (Figure 1). Therefore, features of RNAi effectors are proposed to be similar to cellular miRNAs but not compete detrimentally with it [12]. This might be avoided by strategies using tissue-specific RNA Pol II [45] or weaker Pol III promoters [12].

**Figure 2:**
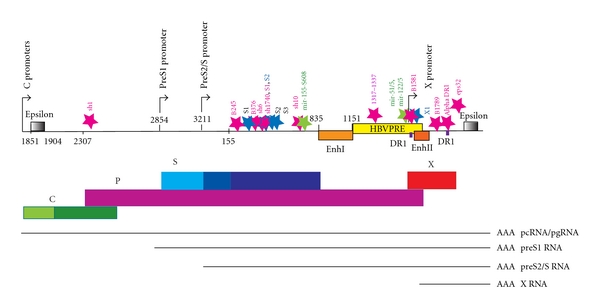
A linear depiction of the HBV genome indicating successful RNAi target sites. Four main promoters (C, S1, S, and X promoters) and regulatory elements such as epsilon, EnhI, EnhII, PRE, and DR1 are indicated on the HBV genome. The four overlapping HBV ORFs are indicated as coloured solid boxes. Thin line represents HBV transcripts. The common poly(A) site is represented as “AAA.” Pink stars indicate Group I successful RNAi target sites, while green and blue stars represent Group II and Group III, respectively. The authors designated names of each of the RNA effectors indicated. Numbering indicates nucleotide position, using EcoRI-based numbering system from HBV Genotype A (AM282986, [6]).

A complementary rational design is currently proposed to target conserved regions of the HBV genome. This should minimize viral escape that may occur due to selection pressure of RNAi on the target site to mutate [7, 10, 13, 14]. Surprisingly, these include several highly conserved HBV genomic regions that have been demonstrated to be effective target sites for shRNAs despite the presence of known secondary structures (Epsilon, PRE, Figure 2). These structures were predicted to reduce the effectiveness of RNAi [7, 14]. 

## 5. HBV Genomes to Be Targeted

The HBV genome contains multiple overlapping DNA, RNA, and protein coding features, meaning that any particular RNAi target sequence is likely to be in more than one transcript. The genome is a partially double-stranded circular DNA of 3.2 kb that contains four primary open reading frames (ORFs): the core (C), polymerase (P), surface (S), and X, although there may be other protein products [41, 46]. These ORFs partially overlap each other and are all encoded on the positive strand [47]. Transcription of HBV RNA is initiated by four major promoters—the basal core promoter (BCP), pre S1, preS2/S, and X (Figure 2). These promoters give rise to transcripts that are synthesised in the same direction by host RNA polymerase II. Five major HBV transcripts are known, all are translated. Two sets of C transcripts are initiated at different sites of the BCP promoter. The longest transcript is the 3.6 kb precore mRNA (pcRNA). The shorter C transcript is a pregenomic mRNA (pgRNA) which encodes the C protein (nucleocapsid protein) and the P protein. The other three transcripts are preS1, preS2/S, and X, encoding for S proteins (large surface proteins or preS1), and shorter S proteins (middle and small S proteins or preS2 and S) and the X protein (a transcriptional transactivator), respectively (Figure 2). Therefore, the HBV genome is highly compact and HBV genes are arranged in such a way that many sequences have multiple roles. 

Although this compact arrangement restricts plasticity and limits the ability of the virus to mutate, HBV has significant diversity among HBV genotypes [48, 49] and HBV genomes exist as quasispecies in cells. With drugs targeting HBV polymerase (such as lamivudine, adefovir, and an acyclic nucleoside phosphonate), emergence of HBV-resistant mutants develops during treatment [50]. An escape mutant was also selected for during shRNA treatment, discussed later [10].

## 6. Successful RNAi Strategies against HBV

Several RNAi effectors successfully downregulate HBV gene expression and replication in differing assay systems. A “very highly active” benchmark of >95% reduction of extracellular viral particles from plasmid encoded HBV has been suggested for shRNAs warranting further development [7]. However, different experimental approaches and assays make quantitative comparison difficult. Assays for RNAi inhibition commonly used are (i) reporter gene assays, for example luciferase [14], (ii) reduction of viral RNAs from HBV derived from a plasmid in cultured cells [7], (iii) HBV-expressing transgenic mice or cells [12, 51], and (iv) hydrodynamically HBV-infected mice [9]. 

Analysis of characteristics of successful targets revealed different strategies of rational design for RNAi effectors, RNAi approaches, and mechanism of delivery. Nevertheless, these could be classified into 3 main groups: Group I: sequence conservation-based rational design-shRNA expression vectors (Pol II/III promoter) using a liposome delivery method; Group II: sequence conservation-based rational design-miRNA expression vectors (Pol II promoter) using a liposome delivery method; Group III: single siRNA programme prediction-shRNA expression vectors (Pol III promoter) using viral vector delivery methods (reviewed in [8, 9]). Successful target sequences and RNAi inhibitory effects of these 3 groups are indicated in Table 1. A summary of the effective target positions is shown in Figure 2.

## 7. Variation in HBV Genotypes

The 3.2 kb HBV genome is classified into eight main genotypes (A–H) with over 8% sequence diversity, with genotypes A–D the most prevalent [13, 48, 49]. I is newly discovered but not ratified [54]. Therefore it is not surprising that there are few regions conserved across all genotypes [7, 13]. These regions are often in sites of functional conservation in the RNAs or DNA, including the epsilon RNA and enhancer DNA elements (Figure 2). Some are in sites of overlapping genes, where the two open reading frames constrain sequence. Sun et al. [7] identified only one sequence of 17 bases conserved across representatives of all genotypes (1181–1897). They therefore used lesser stringency criteria of ≥98% or ≥95% identity for ≥15 or more bases across genotypes A–D as a practical limit to identify likely RNAi targets, this being about ~300–500 bases of the genome. They targeted 19 conserved sites in genotype D (ayw) with part of a panel of 21 shRNAs. Many of these were effective (Table 1), including some within the structured RNA epsilon element. The most effective target site in genotype D from that series (sh10) overlaps a conserved block but is not the most conserved target (1 variation in A, B, E; 2 in G, H). However, sh6, another very highly active shRNA, targets a block with no variation in genotypes A–H (Table 1). In other reported studies that included conservation in design, some target better genotype A–C [14]. 

Zhang et al. identified 40 shRNA targets with conservation between genotypes A–I using an alignment of 327 representative sequences from Genbank as a guide [13]. They tested the shRNA against genotypes A–D, and I. The most effective four (B245, B376, B1581, and B1789) were able to reduce HBV production by up to 90% in both *in vitro* transfection and *in vivo* hydrodynamic model systems (Table 1). 

As there was some dissimilarity in target design, there is not good concordance between the targets chosen in the studies of Zhang et al. [13] and Sun et al. [7]. However, some of the best sites had similar sites in the complementary study, but for these there was not good concordance in degree of inhibition. For example B245 (245–265), the best target from Zhang et al., is similar to sh4 (247–257) one of the weakest in Sun et al. Conversely the effective sh6 (416–434) is similar to B415 (415–435) but B415 had only a weak inhibitory effect. This may reflect subtle differences in targets, the vectors or assays used, and supports a need for common standards within experiments as suggested by Sun et al. 

## 8. Rare Variants—Could These Be Selected for by RNAi?

HBV polymerase has a high error rate producing many mutants—most of these with lower replication fitness. There are over 2,500 full-length HBV genomes in the Genbank database, and other databases contain rare sequence variations and mutations [50, 55, 56]. These may represent true replication competent variants, rare nonfunctional RNAs in the infected cells, or PCR or sequencing errors. As it is difficult to distinguish between these possibilities, redesigning an RNAi strategy to avoid them would be difficult. 

In the recent study by Sun et al. they also tested the several shRNAs designed for HBV against Wooley monkey HBV (WMHBV). In WMHBV target sites differed in 1–4 positions. Some single variations retained partial activity (e.g., sh6 still inhibited to 14%), but most abolished it, as did single mutations in the shRNA [7]. This is consistent with the idea that there must be an exact match between shRNA and target. However, it might be expected that variation in the 5′ end of the target, as was the case with sh6, would be more tolerated based on other RNAi studies, but this has not been systematically tested for HBV targets. 

One study has found a resistant mutation that could be selected for following shRNA treatment in cell culture [10]. The shRNA used was designed to target a conserved site in all except genotype H (456–476) and was found to be effective in A–C. However a rare mutation in genotype C could be selected for by shRNA treatment in cultured cells. This mutation was silent with respect to both S and Pol overlapping protein coding and found in only one chronic carrier. Emergence of this type of shRNA-induced resistance has been seen for other viruses, notably HIV [57, 58]. In some cases like this a redundant pool of shRNAs containing a mix at a single position might be effective, for example, where a single position was changed, T472C or T472G in genotype H [10]. To our knowledge this approach has not been used for HBV variants. 

Deep sequencing using next (or new) generation (NGS) sequencing technologies allows the sequencing of many members of the HBV quasispecies infecting a single human. For HBV several studies have been done to investigate the emergence of mutations due to drugs targeting proteins [59–61]. Low prevalence drug resistance mutations could be detected by NGS with a greater sensitivity than PCR in both naïve and treated patients. These initial studies focused on mutations that change one of 288 RT amino acids. Many novel changes were detected—71 present in over 1% of the 2,800–18,000 sequences from each patient. Data from such deep sequencing studies would also be useful in design of RNAi against conserved sites, if possible rare tolerated mutations or polymorphisms should be avoided in target sites. 

## 9. Conclusion and Directions for Further Studies

Most current assays for HBV replication more closely mimic acute HBV infection, with a single infecting genotype. In chronic infection the system where the virus is represented by a quasispecies in the infected individual selection due to the RNAi might be different [13]. 

It is possible that the rare genotypes, for example H, would require a genotype-specific RNAi combination. Highly effective RNAi that does not target conserved blocks would still be useful if the genotype of the target is known. 

Rationally designed RNAs, targeted in combinations [40, 62–64], delivered by “state-of-the-art” vectors could be an effective anti-HBV treatment [4, 5, 8, 9]. Such design strategies would need to take into account conservation in the HBV genome. Several groups have identified effective target sites that are beginning to fulfill these criteria, and these will provide tools for further development. 

## Figures and Tables

**Table 1:** Successful RNAi strategies targeting HBV utilising genomic conservation.

Group	Design of the RNAi effector	RNAi approach	Experimental system	Nucleotide position*	Designated name	Target sequence	RNAi effect (% reduction)	Ref.
				416–434	Sh6	CTGCTATGCCTCATCTTCT	95% (encapsidated pgRNA), 98% (DNA)	
				458–476	1740 SLAS	GGTATGTTGCCCGTTTGTC	92% (encapsidated pgRNA), 96% (DNA)	
				720–738	Sh10	CTGTTTGGCTTTCAGTTAT	95% (encapsidated pgRNA), 98% (DNA)	[7]
				1823–1841	DR1	TTTCACCTCTGCCTAATCA	96% (DNA)	
				1848–1867	Eps32	TTCATGTCCCTACTGTTCAA	96% (DNA)	
I	Sequence conservation among HBV genotypes	shRNA expression vector (Pol II/III promoter)	Liposome delivery/human liver cell line	2421–2439	Sh1	GTCGCAGAAGATCTCAATC	86% (encapsidated pgRNA), 96% (DNA)	
				458–476	S1	GGTATGTTGCCCGTTTGTC	90% (total RNA, DNA)	[10]
				1317–1337	PRE1317	AAAGCTCATCGGAACTGACAA	80% (cccDNA)	[14]
				245–265	B245	AGTCTAGACTCGTGGTGGACT	~80% (pgRNA, pcRNA), 90% (DNA)	
				376–396	B376	GATGTGTCTGCGGCGTTTTAT	~80% (pgRNA, pcRNA, DNA)	[13]
				1581–1601	B1581	GCACTTCGCTTCACCTCTGCA	~70% (pgRNA, pcRNA), 80% (DNA)	
				1778–1798 (sic)	B1789	AGGCTGTAGGCATAAATTGGT	~70% (pgRNA, pcRNA) 80% (DNA)	

II	Sequence conservation among HBV genotypes	miRNA expression vector (Pol II promoter)	Liposome delivery/human liver cell line	761–782	Mir-155-S608	CCAAGTCTGTACAGCATCGTGA	80% (HBs mRNA)	[52]
				1575–1599	Mir-51/5 Mir-122/5	CCGTGTGCACTTCGCTTCACCTCTG	90% (HBsAg)	[24]

III	Analysis of a single genotype (representative examples)	shRNA expression vectors (Pol III promoter)	Viral vectors/human liver cell line	456–476	S1	AAGGTATGTTGCCCGTTTGTC	90% (Total RNAs)	[40]
				1644–1664	X1	AAGGTCTTACATAAGAGGACT	90% (Total RNAs)	
				322–342	S1	AACCTCCAATCACTCACCAAC	80% (HBsAg)	
				540–560	S2	AAGGAACCTCTATGTATCCCT	80% (HBsAg)	[53]
				589–609	S3	AAATTGCACCTGTATTCCCAT	80% (HBsAg)	

*indicates *Eco*RI site-based numbering systems using Genotype A (AM282986) as described in [6].
